# Motivation and Second Language Pragmatics: A Regulatory Focus Perspective

**DOI:** 10.3389/fpsyg.2021.753605

**Published:** 2021-10-29

**Authors:** Yiran Zhang, Mostafa Papi

**Affiliations:** ^1^Jing Hengyi School of Education, Hangzhou Normal University, Hangzhou, China; ^2^School of Teacher Education, College of Education, Florida State University, Tallahassee, FL, United States

**Keywords:** L2 pragmatics, motivation, regulatory focus, promotion, prevention

## Abstract

This study investigates how learners’ chronic motivational characteristics, that is their regulatory focus (Higgins, 1997), can account for differences in L2 pragmatic production in general and across situations with different levels of power, social distance, and imposition. One-hundred-twenty-one L1-Mandarin learners of English as a second language completed a regulatory focus questionnaire and a discourse completion task focusing on two types of speech acts: request and opinion. Multiple regression results showed that learners’ promotion focus, concerned with advancement, growth, accomplishments, positively predicted their pragmatic production in general, and especially in situations where the learner is subject to a higher degree of imposition, has lower power and is socially distant from the interlocutor. On the other hand, the prevention focus, which is concerned with safety, security, and calmness, negatively predicted pragmatic production, especially in those situations. The findings provide support for the role of motivational dispositions in the level of learners’ L2 pragmatic competence. Theoretical and instructional implications are discussed.

## Introduction

Second language (L2) pragmatics is one of the crucial areas in second language acquisition (SLA) research. Lack of L2 pragmatic knowledge and the ability to use the language properly can affect the efficiency and quality of the communication, and cause misunderstandings. Appropriate language use in different sociolinguistic and sociocultural contexts is regarded as one of the core abilities within the framework of communicative competence (Canale and Swain, 1980). Various models of communicative competence (Bachman, 1990; Bachman and Palmer, 1996, 2010) have explicitly positioned pragmatic competence as a central component of L2 ability, along with grammatical, discourse, and strategic competencies. These models emphasize the importance of the sociocultural conventions and norms of language use in L2 learning. Other than learner-external factors such as experience in target language community (e.g., Bardovi-Harlig and Dörnyei, 1998; Niezgoda and Rover, 2001; Taguchi, 2008), and pragmatic instruction (for review see Taguchi, 2015) that have found to influence L2 pragmatics, studies have been conducted to identify individual learner factors contributing to L2 pragmatic competence. These studies have shown the importance of the role of personality (e.g., Verhoeven and Vermeer, 2002; Kuriscak, 2010; Taguchi, 2014), cognitive variables (e.g., Taguchi, 2008), and proficiency (e.g., Rose, 2000; Félix-Brasdefer, 2007; Taguchi, 2007, 2011a, b; Bella, 2014) in L2 pragmatic learning and development. Focusing on Chinese ESL and EFL learners, factors such as proficiency (e.g., Wei, 2011) and study abroad experience (e.g., Ren, 2012, 2014) have been found to affect their pragmatic competence. By analyzing the speech samples of college learners of English in China, Wei (2011) found that learners with higher proficiency levels were more active and involved than intermediate ones in the use of pragmatic markers and showed a better sensitivity to different types of interactive context. Ren (2012) examined the effects of study abroad environment on the pragmatic development of Chinese speakers by comparing two groups of students (study abroad vs. at home). Although no significant benefit of study abroad has been found for their development of refusal strategies, the study abroad experiences generally influenced Chinese speakers’ language use and sociopragmatical choices. His later longitudinal study, which investigated the cognitive processes of learners during their study abroad by using the retrospective verbal report (Ren, 2014), further confirmed the positive influence on their pragmatic production. The learners of the study reported decreases in pragmatic difficulties and increases in pragmatic knowledge over the course of one academic year.

Motivation, as one of the “big two” individual difference factors along with aptitude in SLA research, however, has not attracted enough interest in the field of L2 pragmatics. A few empirical studies have examined the effects of motivation on pragmatic learning (e.g., Takahashi, 2005, 2015; Tajeddin and Moghadam, 2012; Yang and Ren, 2020). These studies have generally shown that L2 learners who were intrinsically motivated and had stronger communication-oriented motivation were more likely to have a better pragmatic competence. Whereas, these studies suggest a role for motivation in L2 pragmatics competence, the link between chronic (trait-like) motivational dispositions and qualitative differences in learners’ pragmatic competence has remained underexplored. In fact, motivation has often been used only as a ***post hoc*** explanation for the contradictions and mixed findings in the L2 pragmatics literature (e.g., Cook, 2001; Niezgoda and Rover, 2001) rather than an important factor in learners’ level and development of L2 competence.

This has probably been mainly due to what Papi (2016, 2018) has argued to be a “quantity perspective” toward the study of motivation. Highlighting the lack of research linking different dimensions of L2 competence and motivation, Papi (2018) argued that “SLA researchers have predominantly approached motivation as a quantity of energy that is produced to initiate, continue, and complete the learning pursuit” (p. 707), but have failed to examine it as a factor that could result in qualitative differences in learners’ goals, behaviors, and learning outcomes. Following Papi’s (2016, 2018) call for exploring the connection between chronic motivational dispositions and learners’ L2 characteristics, the present study employs Higgins’s (1997) motivation theory to see how learners’ chronic regulatory focus (i.e., promotion and prevention) predicts qualitative differences in their L2 pragmatic competence. According to Papi et al. (2019), learners with a chronic promotion focus, concerned with accomplishments, advancement, and growth, use an eager strategic inclination in language learning which maximizes their use of the target language; those with a prevention focus, who are concerned with safety, stability, and calmness, on the other hand, use a vigilant strategic inclination in their language learning in order to minimize the possibility of making mistakes and avoid potential negative consequences. Given the amount of input and opportunities of engagement in social interactions are essential for developing L2 pragmatics competence, this study seeks to explore how learners with different regulatory foci, which have been found to lead to differences in learners’ strategic inclinations in L2 use (Papi et al., 2019; Papi and Khajavy, 2021), vary in terms of their L2 pragmatic production, which refers to their appropriateness of using English as a second language in different social contexts.

## Literature Review

### Motivation and L2 Pragmatics

Motivation has been an important topic of research in the field of applied linguistics over the last five decades. Numerous studies from several theoretical perspectives have provided empirical evidence for the important role of motivation in language learning (see Dörnyei and Ushioda, 2021). Nonetheless, the topic has been examined in relation to L2 pragmatics only in a few studies.

Takahashi (2005) investigated the effect of motivation and proficiency on English as a foreign language (EFL) learners’ ability to recognize target request expressions in written dialogues. Participants completed a motivation questionnaire and an oral task on English request forms after receiving an implicit instructional treatment on the target pragma-linguistic features. Results showed that learners with more intrinsic motivation noticed more target forms and had more awareness of target pragmatic forms than less-motivated learners. In another study on the effects of learner profiles on pragma-linguistic awareness and learning, involving 154 Japanese EFL learners, Takahashi (2015) also found that learners with stronger communication-oriented motivation noticed more bi-clausal request forms (e.g., I am wondering); however, motivation was not associated with L2 pragmatic production. Tagashira et al. (2011) investigated how Japanese EFL learners’ patterns of motivation influenced their pragmatic awareness. A total of 162 intermediate-level EFL learners in a Japanese university completed a questionnaire measuring their motivation as well as pragmatic and grammatical awareness. The results showed that motivation accounted for differences in recognition of pragmatic errors, but not grammatical errors. Additionally, the more self-determined or intrinsically motivated learners showed a better perception of the appropriateness of the utterances. Tajeddin and Moghadam (2012) investigated EFL learners’ general and speech-act-specific motivation (motivation for using requests, refusals, and apologies) on one hand, and their performance on the specific speech acts using a written discourse completion task (DCT) on the other hand. Results showed a significant impact of speech-act-specific motivation on learners’ speech act production, while the general pragmatic motivation did not predict learners’ production significantly. Concentrating on L1-Mandarin Chinese speakers, Yang and Ren (2020) investigated the relationship between Chinese EFL learners’ L2 motivation and their pragmatic awareness by adopting the L2 Motivational Self System (L2MSS) questionnaire (Taguchi et al., 2009). The results showed that the intended learning efforts, attitudes toward the L2 community, and attitudes toward learning English significantly predict levels of pragmatic awareness. However, there was no significant correlation found between pragmatic awareness and ideal/ought-to L2 self.

In sum, these studies show that learners with stronger intrinsic or communication-oriented motivation noticed more target pragmatic forms, had better awareness for L2 pragmatic forms (Takahashi, 2005, 2015), and showed better recognition of pragmatics errors (Tagashira et al., 2011; Tajeddin and Moghadam, 2012). However, they did not show better production of L2 pragmatic forms (Takahashi, 2005, 2015) and did not show better recognition of grammatical errors (Tagashira et al., 2011; Tajeddin and Moghadam, 2012). The studies have all looked at motivation from a limited perspective that assumes higher motivation results in higher pragmatic competence. Whereas there is some truth to the argument, this perspective does not explain, for example, why the motivated learners only have better pragmatic awareness but not production, and why they recognize pragmatic but not grammatical errors. This is mainly because the motivation theory used in almost all the reviewed studies has been an L2-specific version of the self-determination theory (Deci and Ryan, 1980), which posits that the more self-determined the motive is the higher the learners’ motivation to increase their pragmatic knowledge would be. Yang and Ren (2020) attempted to measure the motivation under the framework of L2MSS, their motivational variables (i.e., intended learning efforts, attitudes toward the L2 community, and attitudes toward learning English) generated from a factor analysis, however, still fell within the realm of the self-determination theory. Whereas, this theory can highlight important differences in the extent learners engage in the use of the second language and learning pragmatic skills, it does not explain why some learners are intrinsically motivated in such engagement whereas others are not. Thus, we will employ regulatory focus theory, which outlines chronic motivational systems underlying human preferences and has the potential to account for such inter-individual differences in learners’ L2 pragmatic competence.

### Regulatory Focus Theory

Higgins’ regulatory focus theory (1997) highlights two distinct but coexisting motivational systems that regulate individual’s goal-directed behaviors: the promotion system and the prevention system. In the promotion system, which is characterized by a preoccupation with achieving positive outcomes, goals are perceived as hopes and aspirations. Individuals with a predominant promotion focus are concerned with accomplishments, advancement, and growth. On the other hand, in the prevention system, which is characterized by a preoccupation with avoiding losses, goals are perceived as duties, responsibilities, or obligations. Individuals with a predominant prevention focus are concerned with security, safety, and stability. According to Crowe and Higgins (1997), the promotion and prevention foci also reflect different strategic tendencies in achieving goals. Individuals with a promotion focus are more likely to have an eager strategic tendency in their goal pursuit (Crowe and Higgins, 1997) to ensure that they maximize their opportunities for achieving gains even though there are risks of committing errors (Scholer et al., 2010). On the other hand, individuals with a prevention focus are more likely to have a vigilant strategic tendency (Crowe and Higgins, 1997), to ensure they minimize their losses, and try to be more careful to avoid wrong choices and errors. Thus, the promotion and prevention foci represent two qualitatively different chronic motivational systems distinguished in terms of the goals that motivate individuals (growth vs. security) as well all the strategic tendencies (eager vs. vigilant) they use to achieve their goals.

In the field of applied linguistics, regulatory focus theory has often been used in the form of future L2 self-guides, which have been outlined in the theoretical foundation of Dörnyei’s (2009) L2 motivational self-system and other models of future L2 self-guides (e.g., Teimouri, 2017; Papi et al., 2019). Ideal L2 self, representing one’s image of the L2 user they want to be in the future, has a promotion focus whereas ought L2 self, representing one’s perception of their obligations and responsibilities, has a prevention focus. Ideal L2 self has been found to predict promotion-related behaviors such as eager tendencies to use the second language (Papi et al., 2019) and willingness to communicate in the target language (e.g., Khajavy and Ghonsooly, 2017) and the promotion-related emotion of L2 joy (e.g., Teimouri, 2017). On the other hand, ought L2 self has been found to predict vigilant-related behavior such as low participation in classrooms (Papi and Abdollahzadeh, 2012), vigilant tendencies to use the target language (Papi et al., 2019), and the prevention-related emotion of L2 anxiety (Papi, 2010). Two studies have directly employed the regulatory focus theory. Jiang and Papi (2021) found that a promotion focus negatively predicts L2 speaking anxiety among Chinese learners of English. In Iran, Papi and Khajavy (2021) found that a promotion focus positively predicted ideal L2 selves, which in turn contributed to the emotion of L2 enjoyment, an eager L2 use inclination, and L2 achievement. On the other hand, a prevention focus negatively predicted ought L2 selves, which in turn positively predicted L2 anxiety and vigilant L2 use. The findings suggest that learners with a stronger promotion focus pursue ideal L2 selves, enjoy the learning process, eagerly use the target language, and have higher achievement than others. Those with a stronger prevention focus, on the other hand, are better at meeting obligations and duties, experience more anxiety, are cautious in L2 use, and show poorer achievement than others.

Given the different characteristics and behavioral patterns associated with the promotion and prevention focus, the present study hypothesizes possible connections between L2 learners’ regulatory focus and their L2 pragmatic learning behavior and outcomes. L2 learners with a promotion focus are speculated to take advantage of every opportunity they get to use the target language due to their eager strategic inclinations (Papi et al., 2019; Papi and Khajavy, 2021) and risk-taking tendency (Scholer et al., 2010); they may also pursue maximal L2 learning goals (ideal L2 selves) such as advanced proficiency, which could further motivate their eager behaviors contributing to their pragmatic competence. On the other hand, L2 learners with a prevention focus, who are more risk-averse and have vigilant strategic inclinations, are speculated to have lower levels of pragmatic competence due to their tendency to minimize the possibility of making errors by avoiding the unnecessary use of the L2; they are also motivated by minimal goals (ought L2 selves) such as meeting institutional obligations, which further increases their anxiety (Jiang and Papi, 2021; Tahmouresi and Papi, 2021) and dissuades them from using the target language.

In addition, this study examines how the learners’ performance differs across scenarios of expressing requests and opinions with high and low levels of imposition, power difference, and social distance. Brown and Levinson (1987) identified the three main contextual variables which, combined together, influence how speakers perform politeness. Degree of Imposition (I) is about the cost to the hearer when the speaker makes requests. For example, asking for a large amount of money has a high degree of imposition (I+) whereas asking for a spare pen is low in imposition (I-). Social Power (P) is about the hierarchical relationship between the speakers. In school situations, for instance, teachers have higher power (P+) than students (P-). Social Distance (D) is about how well the interlocutors know each other. For example, when we interact with our friends, social distance is low (D-), while the distance is high when talking to a stranger (D+). Based on these factors, as well as situations that normally happen in school settings, we used scenarios that were either low (IPD-) or high (IPD+) degree of imposition, power imbalance, and social distance between the interlocutors. For example, asking a classmate for a piece of paper, and giving an opinion about a friend’s new shoes is considered IPD- whereas expressing an objection to a professor about a grade is considered IPD+. It is expected that learners with different regulatory focus perform differently in social situations with different degrees of power, imposition, and social distance. Learners with a dominant promotion focus are expected to perform better in social situations where they have lower power status than their interlocutor and the degree of imposition is higher given they tend to see that as an opportunity for a gain whereas those with a prevention focus, who may see the power imbalance and high degree of imposition as a high-risk situation, are expected to perform better in the situation in which they have equal power with their interlocutor. Prevention-focused learners are also expected to perform better than promotion-focused learners in situations where the social distance with the interlocutor is smaller, which suggests less risk involved in the interaction. In sum, promotion-focused learners are anticipated to do well in situations involving higher degrees of imposition, higher power status, and higher social distance (IPD+), whereas prevention-focused learners are expected to show better pragmatic competence in situations involving lower degrees of imposition, equal power with the interlocutor, and smaller social distance (IPD-). The current study, thus, has been guided by the following research questions:

1. What is the relationship between second language speakers’ regulatory focus and their L2 pragmatic production?
  **H1:** *L2 speakers*’ *promotion but not prevention focus will positively predict their L2 pragmatic production.*
2. What is the relationship between second language speakers’ regulatory focus and their L2 pragmatic production in different social situations?
  **H2:** *L2 speakers*’ *promotion focus will positively predict their pragmatic production in IPD+ situations (high degree of imposition, high power, and large social distance), whereas their prevention focus will positively predict their pragmatic production in IPD- situations (low degree of imposition, equal power, and smaller social distance).*

## Materials and Methods

### Participants

The participants in this study were 121 Chinese international students (71 females and 50 males) who speak English as a second language at a large university in the United States. Their age ranged from 19 to 37 (Mean = 26.63, *SD* = 3.80). The majority of the participants were pursuing graduate degrees (*N* = 106) while a minority (*N* = 15) were in undergraduate programs. To minimize and control for the influence of the first language on L2 pragmatics, and highlight the motivational factors, participants with the same first language, Mandarin Chinese, were recruited. Based on the Test of English for International Communication (TOEIC) “Can do” Guide (The Chauncey Group International, 2000), the mean score for the participants’ self-rated English proficiency was 4.07 (*SD* = 0.56) on a scale ranging from 1 (Beginner) to 5 (Advanced), and their length of residence in the United States ranged from 3 months to more than 5 years (*M* = 3.35, *SD* = 1.76). Table 1 displays the detailed demographic characteristics of the participants in the current study.

**TABLE 1:** Summary of participants’ descriptive demographics.

Demographic category	*N* = 121
**Age**	
<21	3 (2.5%)
21–25	45 (37.2%)
26–30	56 (46.3%)
31–35	13 (10.7%)
>35	4 (3.3%)
**Gender**	
Male	50 (41.3%)
Female	71 (58.7%)
**Degree in process**	
Bachelor’s degree	15 (12.4%)
Master’s degree	45 (37.2%)
Specialist degree	4 (3.3%)
Doctoral degree	55 (45.5%)
Postdoctoral degree	2 (1.7%)
**Length of residence in the United States**	
<1 year	21 (17.4%)
1–2 years	26 (21.5%)
2–3 years	22 (18.2%)
3–4 years	20 (16.5%)
4–5 years	6 (5%)
>5 years	26 (21.5%)

### Instruments

#### Regulatory Focus Questionnaire

We used the composite regulatory focus questionnaire developed by Haws et al. (2010) to examine the participants’ chronic regulatory focus. There were 10 items describing specific events that actually occur or have occurred in their life using five-point Likert scales in the questionnaire (1 = strongly disagree, 2 = disagree, 3 = neither agree nor disagree, 4 = agree, 5 = strongly agree). Five items measured the promotion focus (e.g., *Q4: I frequently imagine how I will achieve my hopes and aspirations*) and the other five items measured the prevention focus (e.g., *Q6: Growing up, I usually obeyed rules and regulations that were established by my parents*). The scoring of participants’ regulatory focus in the current study followed the same scoring rubric of Haws et al. (2010); we added up (range: 5–25) the scores for the promotion and prevention items separately. The higher an individual scores on each of the scales, the stronger the respective regulatory focus would be.

#### Discourse Completion Task

To measure the participants’ L2 pragmatic production—the ability to deliver intentions appropriately—a discourse completion task (DCT) was used. Although DCT data may not perfectly represent the actual language in real-world conversation, it provides evidence about the collection of speech act strategies that language learners have at their disposal (Taguchi and Roever, 2017) and their pragmatic intuitions. Additionally, DCTs allow control of contextual factors (i.e., power, social distance, and rank of imposition) for examining the level of appropriateness in different situations, which fits the purpose of this study. The current study focused on two types of speech acts expressing requests and opinions. These two were selected based on Garcia’s (2004) analysis of naturalistic conversations in university settings in her corpus-based study of pragmatic utterances. Garcia (2004) analyzed conversations across three registers: conversations among students in study groups, conversations between professors and students during office hours, and service encounter conversations. She found that speech acts of requests and opinions are the most common in the corpora. The current study consulted examples in Garcia’s (2004) corpus data, and the DCT items used in this study were adapted from the studies of Takimoto (2009) and Taguchi (2013) and contain scenarios built to fit the situations that happen in university settings. Those situations are divided into two categories based on the degree of imposition (I), social power (P), and social distance (D).

The DCT used in the current study included eight items (four IPD– and four IPD+) with scenarios triggering the participants to either share their opinions (four items) or make requests (four items). The questionnaire was delivered through Qualtrics. The participants were asked to write what they would say if they were in a certain situation. Since the task was open-ended, a rubric adapted from Taguchi (2013) for appropriateness rating was employed. Appropriateness was defined as the ability to perform speech acts at the proper level of directness, politeness, and formality. The participants’ responses to the DCT items were scored on a five-point scale (1 = very poor, 2 = poor, 3 = fair, 4 = good, and 5 = very good) based on the descriptions provided in the rubric. The participants would get a zero if there was no response or if they indicated that they would not say anything in the given situation. Four native English speakers (3 females) were recruited as raters. They were all undergraduate students majoring in education in their 20 s. None of them had a background in Applied Linguistics or a related field. After a short training session, which included getting familiar with the rubric, practice rating, and discussion, they first rated the answers individually. Each sample was rated by at least two raters. Interrater reliability, measured using Pearson’s correlation, was high (*r* = 0.90). The samples that had two points difference between two raters were discussed by the raters. A third rater was invited to the discussion if the difference could not be resolved by the two original raters. The average scores were assigned for the items with one-point difference. A higher score represents a more native-like pragmatic production in terms of appropriateness.

#### Demographic Questionnaire

A demographic questionnaire was used to collect the background information from the participants including gender, age, first language, length of residence in the United States, and their self-reported English proficiency level. The self-report proficiency items contained 10, five-point Likert-scale items that were based on the TOEIC “Can do” Guide (The Chauncey Group International, 2000). The original scale included items related to listening, speaking, and interactive skills. Because of the length of the questionnaires, however, the current study picked 10 items only from the interactive skills section. The mean scores of the self-reported proficiency items were calculated and used as the participants’ proficiency measurement. The participants’ length of residence was recorded based on how many years they had spent in the United States.

### Procedures

An email with information about the purpose of the study, the criterion for choosing the target participants, and participation expectations was first sent to Chinese international students studying at a university asking about their willingness to participate in exchange for a $10 gift card. A total of 152 Chinese students in the university replied with their contact information. The questionnaires were then distributed to those respondents through Qualtrics. The participants were allowed to complete the questionnaires at a time and location of their convenience with the permission to pause and resume whenever they wanted. One hundred and twenty-four (124) of the participants completed the questionnaire. Finally, a thank-you email along with the gift card was sent to each individual. From the total of 124 completed participants, three of them were removed from the data as they had spent a significantly short amount of time completing the questionnaires and choosing the same answer for every question.

### Data Analysis

The present study used regulatory foci (promotion and prevention) as predictor variables and pragmatic production measures as the outcome variables. Due to the fact that participants’ self-reported English proficiency and their length of residence in the United States have been found to influence L2 speakers’ pragmatic competence in previous studies (e.g., Bardovi-Harlig and Dörnyei, 1998; Rose, 2000; Niezgoda and Rover, 2001; Taguchi, 2007, 2008, 2011a, b; Bella, 2014), the two factors were also entered as predictor variables in all the regression analyses in order to control for their potential effects. Before conducting data analysis for each specific research question, Cronbach’s alpha reliability analysis for the Composite Regulatory Focus Questionnaire, as well as the proficiency items were conducted. Cronbach’s α coefficient was 0.74 for the promotion scale (*M* = 3.66, *SD* = 0.65), 0.72 for the prevention scale (*M* = 3.46, *SD* = 0.68), and 0.87 for proficiency items (*M* = 4.07, *SD* = 0.56), suggesting the internal consistency of the scales. Pearson’s Correlation analyses between the predictors were also conducted. As can be seen in Table 2, the highest correlation was a medium one between Length of Residence and English Proficiency, suggesting that multicollinearity was not a problem for testing our regression models.

**TABLE 2:** Pearson correlations between predictor variables.

		Mean	Std. deviation	1	2	3
1.	Promotion	3.66	0.65			
2.	Prevention	3.46	0.68	–0.11		
3.	Proficiency	4.07	0.56	0.33**	–0.09	
4.	Length of Residence	3.35	1.76	0.06	–0.11	0.42**

**p < 0.01.

The participants’ pragmatic production was measured using the DCT. To answer the research questions, the task generated three different types of scores as the outcome variables: an overall DCT score (*M* = 3.94, *SD* = 0.35) which represents L2 speakers’ overall productive competence; an IPD+ score (*M* = 3.80, *SD* = 0.49) which represents their productive competence when talking to a person who has higher power, a larger distance, and in a situation of higher imposition; and an IPD- score (*M* = 4.08, *SD* = 0.37) representing the participants’ productive competence in a situation of a lower imposition when talking to a person who has equal power and shares a small distance. The descriptive statistics of the outcome variables are presented in Table 3.

**TABLE 3:** Descriptive statistics of outcome variables.

Variable names	Range	Mean	Std. deviation
DCT score	0–5	3.94	0.35
DCT IPD+	0–5	3.80	0.49
DCT IPD–	0–5	4.08	0.37

*DCT, Discourse Completion Task; IPD, Imposition, Power, and Distance.*

## Results

To answer the first research question concerning the relationship between L2 speakers’ regulatory focus and pragmatic production (see Table 4 for inter-correlations), a multiple regression analysis was conducted using Promotion and Prevention as predictor variables, Proficiency and Length of Residence as covariates, and DCT score as the outcome variable (Table 5). The multiple regression results indicated that the model explained 10% of the variance [*F*_(4, 116)_ = 3.18, ***R^2^*** 0.10, *p* = 0.02] with Promotion (*β* = 0.21, *p* = 0.03) being the only variable that positively predicted L2 speakers’ DCT scores. The Beta value of 0.21 suggests that with an increase of one unit in Promotion there would be an increase of 0.21 units in participants’ DCT score. The other predictors did not significantly contribute to the model. Prevention, however, emerged as a near-significant negative predictor (*β* = -0.17, *p* = 0.061). The Beta value of -0.17 suggests that with an increase of one unit in Prevention there would be a decrease of 0.17 units in participants’ DCT score. The results indicated that Promotion was a significant positive predictor of L2 speakers’ DCT sores whereas Prevention was a near-significant negative predictor of DCT scores. Thus, our Hypothesis 1 which stated that the L2 speakers’ promotion but not prevention focus will positively predict their pragmatic production was confirmed.

**TABLE 4:** Pearson correlations between predictors and pragmatic production measures.

	DCT score	DCT IPD+	DCT IPD-
Promotion	0.24**	0.34**	–0.02
Prevention	−0.20*	−0.24*	–0.08
Proficiency	0.15	0.13	0.11
Length of Residence	0.12	0.10	0.10

DCT, Discourse Completion Task; IPD, Imposition, Power, and Distance. *p < 0.05, **p < 0.01.

**TABLE 5:** Multiple regression results for regulatory focus with DCT score as the outcome variable.

	B	Std. error	Beta	t	Sig.	95% CI
(Constant)	3.70	0.31		11.93***	0.000	[3.08, 4.31]
Promotion	0.11	0.05	0.21	2.17*	0.032	[0.01, 0.21]
Prevention	–0.09	0.05	–0.17	–10.89	0.061	[–0.18, 0.00]
Proficiency	0.02	0.07	0.04	0.37	0.712	[–0.10, 0.15]
LOR	0.02	0.02	0.08	0.77	0.443	[–0.02, 0.05]

R^2^ = 0.10. LOR, Length of Residence; DCT, Discourse Completion Task. *p < 0.05, ***p < 0.001.

To answer the second research question concerning whether regulatory focus can predict L2 speakers’ pragmatic production in IPD+ and IPD- situations, two multiple regression analyses were conducted. When IPD+ score were entered as the outcome variable (Table 6), the regression model explained 16% of the variance [*F*_(4, 116)_ = 5.53, ***R^2^*** 0.16, *p* < 0.001]. In addition, whereas Promotion positively predicted their IPD+ scores (*β* = 0.33, *p* = 0.001), Prevention was a negative predictor (*β* = –0.19, *p* = 0.03). The Beta values suggest that with an increase of one unit in promotion there would be an increase of 0.33 units in participants’ IPD+ score and with an increase of one unit in prevention there would be a decrease of 0.19 units in participants’ IPD+ score. The model with IPD- score entered as the outcome variable (Table 7) explained only 2% of the variance and was not significant [*F*_(4, 116)_ = 0.66, ***R^2^*** 0.02, *p* = 0.62]. In addition, none of the predictors contributed significantly to the model. The results indicated that Promotion positively and Prevention negatively predicted their pragmatic production in IPD+ situations which represented the situations in which the respondent needs to make a request or share opinions that have a higher degree of impositions, with an interlocutor who has higher power and a larger social distance. Thus, the first part of our second hypothesis which sated that the L2 speakers’ promotion focus will positively predict their pragmatic production in IPD+ situations was confirmed. However, in IPD- situations where the participants were asked to perform and recognize in situations that have a low degree of impositions, with equal social power relationships and small social distances between the interlocutors, neither Promotion nor Prevention predicted pragmatic production. Thus, the second part of Hypothesis 2 was not confirmed since the prevention focus did not predict the participants’ pragmatic production in IPD- situations.

**TABLE 6:** Multiple regression results for regulatory focus with DCT IPD+ score as the outcome variable.

	B	Std. error	Beta	t	Sig.	95% CI
(Constant)	3.41	0.43		8.02***	0.000	[2.57, 4.25]
Promotion	0.25	0.07	0.33	3.57**	0.001	[0.11, 0.39]
Prevention	–0.14	0.06	–0.19	−2.24*	0.027	[–0.26, –0.02]
Proficiency	–0.02	0.09	–0.03	–0.25	0.804	[–0.20, 0.15]
LOR	0.02	0.03	0.07	0.72	0.472	[–0.03, 0.07]

R^2^ = 0.16. LOR, Length of Residence; DCT, Discourse Completion Task; IPD, Imposition, Power, and Distance. *p < 0.05, **p < 0.01, ***p < 0.001.

**TABLE 7:** Multiple regression results for regulatory focus with DCT IPD- score as the outcome variable.

	B	Std. error	Beta	t	Sig.	95% CI
(Constant)	4.05	0.34		11.82***	0.000	[3.37, 4.72]
Promotion	–0.04	0.06	–0.06	–0.62	0.535	[–0.15, 0.08]
Prevention	–0.04	0.05	–0.07	–0.77	0.444	[–0.14, 0.06]
Proficiency	0.06	0.07	0.10	0.91	0.363	[–0.08, 0.20]
LOR	0.01	0.02	0.05	0.51	0.610	[-0.03, 0.05]

R^2^ = 0.02. LOR, Length of Residence; DCT, Discourse Completion Task; IPD, Imposition, Power, and Distance. ***p < 0.001.

## Discussion

This study investigated the relations between ESL learners’ motivational characteristics and their L2 pragmatic production. The results show that learners’ promotion focus positively predicted their pragmatic production in general, and especially in situations where the learner is subject to a higher degree of imposition, has lower power, and is socially distant from the interlocutor. The prevention focus, on the other hand, negatively predicted pragmatic production, especially in those situations. These findings are in line with the previous research (e.g., Takahashi, 2005, 2015; Tajeddin and Moghadam, 2012; Yang and Ren, 2020) showing that learners’ motivation serves as an important factor that influences L2 pragmatics. In addition, by focusing on their regulatory focus (Higgins, 1997), the current study outlined chronic motivational systems underlying the learners’ preferences and showed the potential to account for the inter-individual differences in learners’ L2 pragmatics.

### Regulatory Focus and L2 Pragmatics

The promotion focus is concerned with growth and accomplishments (Higgins, 1997; Higgins and Cornwell, 2016). Individuals with a promotion focus are sensitive to the presence or absence of positive outcomes and are motivated by ideal selves. Promotion learners’ focus on the positive, rather than negative, end-states leads them to take more risky actions (Scholer et al., 2010), and use eager strategies to take advantage of different opportunities to approach their goals (Crowe and Higgins, 1997). In second language learning, learners with an ideal L2 self, which has a promotion focus, have been found to be more willing to communicate in the second language (Khajavy and Ghonsooly, 2017; Teimouri, 2017) and have an eager strategic inclination using their second language (Papi et al., 2019). In L2 pragmatics literature, intensity of interaction was found as a significant factor that influence L2 pragmatic development (Bardovi-Harlig and Bastos, 2011). Kinginger (2008) found that the degrees of engagement in L2 community (e.g., native speakers, host families) when studying abroad could affect individuals’ pragmatic competence. Similarly, the intended learning efforts were found as the predictor of L1-Mandarin EFL learners’ pragmatic awareness (Yang and Ren, 2020). Further supporting the findings of the present study, attitudes toward the L2 community and attitudes toward learning English were the other two factors revealed by Yang and Ren (2020) to affect learners’ pragmatic awareness. Promotion learners, as Papi (2018) found in his study, are more likely to have positive attitudes because they are more open to new experiences. Such an eager involvement in the use of the target language which has been found to lead to L2 achievement (Papi and Khajavy, 2021) and contribute to pragmatic development among L2 learners (e.g., Kinginger, 2008; Bardovi-Harlig and Bastos, 2011), appears to have put promotion-focused learners at an advantage in terms of L2 pragmatics competence. In other words, the L2 users with a promotion focus seem to be more willing to take risks, engage in different L2 use opportunities, and, as a result, be more exposed to and better learn the socio-cultural and pragmatics dimensions of the target language.

Individuals with a predominantly prevention focus, on the other hand, are motivated by obligations, responsibilities, and duties (Higgins, 1997; Higgins and Cornwell, 2016) and their sensitivity to the presence or absence of negative outcomes makes them more risk-averse (Scholer et al., 2010), and vigilant in their goal pursuit (Crowe and Higgins, 1997). In L2 learning, the ought L2 selves, which have a prevention focus and represent learners’ obligations and responsibilities, have been found to result in the prevention-focus emotion of anxiety (e.g., Papi, 2010; Teimouri, 2017), lower participation in classroom activities (Papi and Abdollahzadeh, 2012), and a vigilant strategic inclination concerned with the minimal use of the target language in order to avoid making mistakes and its negative consequences (Papi et al., 2019). Such a vigilant approach in the use of the target language is likely to have led learners with a prevention focus to avoid engaging in different opportunities for the use of the target language, which, in turn, has led to their lower L2 pragmatics knowledge and competence (Kinginger, 2008; Bardovi-Harlig and Bastos, 2011). The connection between such dispositional characteristics and pragmatics has also been highlighted in a study by Kuriscak (2010) who found that learners with a higher extraversion, representing promotion-related characteristics such as being sociable and talkative, had better pragmatic production than learners with a higher level of neuroticism, representing prevention-related attributes such as emotional instability and anxiety.

### L2 Pragmatics in Different Social Situations

Promotion-focused individuals are concerned about accomplishments, advancement, and growth and are sensitive to the presence or absence of positive outcomes (Crowe and Higgins, 1997). Before engaging in interaction, promotion-focused learners might think about the possible gains of their engagement in L2 interaction especially in IPD+ situations where they may perceive to be some gain opportunities. These learners may want to impress the person with a higher power, and/or may see the situation as an opportunity to create a bond with someone they are not socially close with or to accomplish a task for the person even though it may require a high level of imposition. Given the promotion-focused learners’ eager inclination to engage in and take advantage of such situations for personal and professional advancement and growth (Papi et al., 2019), they tend to better develop the pragmatic competence required to function effectively in such situations.

L2 speakers with a prevention focus, on the other hand, are concerned with safety and security, and sensitive to the presence or absence of negative outcomes (Crowe and Higgins, 1997). Before making decisions about whether to use the target language, prevention-focused individuals might think about what they would lose if they got involved in the interaction. Prevention-focused learners perceive IPD+ situations, where there is a high degree of imposition, the interlocutor has higher social power, and there is a large social distance between the respondents, to potentially involve a lot to risk. The learners, for instance, maybe concerned about getting rejected due to their mistakes or making a negative impression on someone who has higher power than them or someone they are not socially close with. The failure to properly communicate in this situation may thus result in negative consequences which motivate prevention-focused learners to adopt a vigilant strategic inclination (Papi et al., 2019) and avoid engagement with interlocutors who have higher social power and larger social distance and especially if the interaction could involve high degrees of imposition. Such a vigilant L2 use strategy could possibly have led to fewer opportunities for prevention-focused learners to learn the pragmatic and sociocultural aspects of the target language, hence their less-than-ideal pragmatic production and competence especially in IPD+ situations.

On the other hand, L2 speakers’ promotion and prevention did not significantly predict their pragmatic production in IPD- situations, such as talking to a friend who has equal power as the participants in lower imposition situations. This might be due to the fact that IPD- situations are the default social situations that international students more commonly encounter in their life as students studying at a US university. For example, going to classes is an obligatory context for all students regardless of their regulatory focus or motivation. Because in these situations the degree of imposition is low, the social power is equal, and the social distance is small, the potential costs and values associated with interacting in these situations may also be low and not make a difference between learners with different regulatory focus.

These findings support Papi’s (2016, 2018) proposal that understanding the role of motivation in second language learning can be best achieved through a motivation-as-quality approach. L2 learners’ success in achieving the highest levels of pragmatics competence is thus not only a function of how motivated the learners are but also influenced by the chronic motivational differences that learners internalize throughout their lives. Considering such motivational dispositions can thus help us shed light on the inter-individual differences in L2 pragmatics competence.

## Conclusion

The current study adopted regulatory focus theory (Higgins, 1997) in order to investigate how English learners’ chronic motivational characteristics lead to differences in their L2 pragmatic competence and how the effect varies across situations with different levels of power, social distance, and imposition. The results of the study confirmed our expectations by showing that L2 speakers’ promotion regulatory focus positively predicted their pragmatic production in general and especially in IPD+ situations, where the learner has lower power, is socially distant from the interlocutor, and there is a higher degree of imposition; the prevention regulatory focus, on the other hand, negatively predicted pragmatic competence especially in IPD+ situations. These findings suggest that due to their eager and risk-taking nature of their strategic inclinations in goal pursuit, L2 speakers with promotion focus are more likely to take risks and engage in interaction with L2 speakers especially in IPD+ situations where the learners perceive the potentials for accomplishments, advancement, and growth to be maximal. L2 speakers with a prevention focus, on the other hand, adopt a risk-averse vigilant strategic inclination in order to minimize their possibility of making errors and facing negative consequences; therefore, they avoid the interaction opportunities which could otherwise contribute to their pragmatic competence in such situations.

## Limitations and Future Directions

The data for the current study were collected from international students who speak Mandarin Chinese as their first language (L1) and are currently English as a second language speakers at a university in the United States. Although the hypotheses and assumptions were mostly confirmed as expected, the results might not be generalized to other groups with different L1 backgrounds, in different language learning contexts (for example, EFL contexts), or at different stages of language learning. Additionally, it will also be interesting to examine other aspects of pragmatic competence such as speech styles, and interactional features that routinely occur in a conversation. Such expansion on the construct will expand the scope of literature on the role of individual characteristics in L2 pragmatic learning. The DCT items adapted from Takimoto (2009) and Taguchi (2013) were kept intact as much as possible to maintain validity. However, this may possibly cause the items to be mismatched to the experiences of the participants of the current study. The production data gathered through the DCT to measure L2 pragmatics in the current study were non-interactive, meaning that the participants were not required to interact with an interlocutor and only needed to produce one-way responses. Such production data are easier to control and analyze but may not exactly replicate real-world interactive situations. Employing more interactive tasks and methods such as role-plays, elicited conversation, and natural interaction can reach better approximations of actual pragmatic performance. The current study explained the links between motivational orientation and L2 pragmatic production by connecting the findings from L2 pragmatics literature and learners’ chronic motivational characteristics. Based on our findings, we argue that the roles of L2 speakers’ regulatory foci in L2 pragmatic development through learners’ eager and vigilant strategic inclinations cannot be neglected. Future studies can directly examine those possible strategic mediators. It would also be interesting to use qualitative methods such as interviews, think-alouds, and observation to more deeply explore L2 learners’ motivated behaviors, strategies, and perceptions in relation to pragmatics learning. Finally, the current study was based on single-time data collection. To better understand the pragmatics development in relation to motivation, longitudinal studies tracking the development of L2 learners’ pragmatic competence could be eye-opening.

## Implications

The results of the current study provide some significant implications for L2 pragmatics research and instruction. This study introduced a new perspective on motivation that highlights how individuals’ chronic motivational dispositions could possibly influence their strategic inclinations in their L2 learning process, thereby result in different levels of L2 outcomes (Papi, 2016, 2018). The relationship between L2 learners’ chronic regulatory focus and their pragmatic competence provides an explanation as to why individuals who receive similar L2 instruction, spend a similar amount of time in the target language environment, and have a similar level of L2 proficiency, perform differently in terms of pragmatics.

Both teaching and learning can be more effective when L2 learners’ dominant regulatory orientations are reflected in the curriculum, teaching methods, classroom activities, and assessment (Papi, 2016, 2018). The results of the present study suggest that the promotion regulatory focus could contribute to the level of pragmatics competence. Creating a promotion-focused learning environment and adopting a promotion-focused approach that encourages risk-taking, engagements, creativity, and eagerness in teaching L2 pragmatics could thus have positive effects on L2 learners’ learning behavior and outcomes (see Papi and Khajavy, 2021). Promotion-focused instructional and classroom management styles such as minimizing error correction and maximizing learners’ opportunities to talk in the classroom could elicit language learners’ engagement and eagerness, and benefit L2 pragmatics learning. Instead of prevention-focused tasks that require attention to details and vigilance (Van Dijk and Kluger, 2011), promotion-focused tasks that require creativity and risk-taking can elicit L2 use and engagement, and positively contribute to L2 pragmatic learning.

## Data Availability Statement

The raw data supporting the conclusions of this article will be made available by the authors, without undue reservation.

## Ethics Statement

The studies involving human participants were reviewed and approved by the Florida State University. The patients/participants provided their written informed consent to participate in this study.

## Author Contributions

YZ and MP contributed to the conception and design of the study. YZ organized the database, collected the data, and performed the statistical analysis. YZ wrote the first draft of the manuscript. Both authors contributed to the manuscript revision, read, and approved the submitted version.

## Conflict of Interest

The authors declare that the research was conducted in the absence of any commercial or financial relationships that could be construed as a potential conflict of interest.

## Publisher’s Note

All claims expressed in this article are solely those of the authors and do not necessarily represent those of their affiliated organizations, or those of the publisher, the editors and the reviewers. Any product that may be evaluated in this article, or claim that may be made by its manufacturer, is not guaranteed or endorsed by the publisher.
